# Comparative Epigenetic Profiling Reveals Distinct Features of Mucosal Melanomas Associated with Immune Cell Infiltration and Their Clinical Implications

**DOI:** 10.1158/2767-9764.CRC-23-0406

**Published:** 2024-05-28

**Authors:** Jie Dai, Jia Jia, Fanshuang Zhang, Kaihua Liu, Yanfeng Xi, Pei Yuan, Lili Mao, Xue Bai, Xiaoting Wei, Bingning Wang, Jiangtao Li, Yang Xu, Ting Liu, Shuang Chang, Yang Shao, Jun Guo, Jianming Ying, Lu Si

**Affiliations:** 1 Key Laboratory of Carcinogenesis and Translational Research (Ministry of Education/Beijing), Department of Melanoma and Sarcoma, Peking University Cancer Hospital and Institute, Beijing, P.R. China.; 2 State Key Laboratory of Molecular Oncology, Department of Pathology, National Cancer Center/National Clinical Research Center for Cancer/Cancer Hospital, Chinese Academy of Medical Sciences and Peking Union Medical College, Beijing, P.R. China.; 3 Geneseeq Research Institute, Nanjing Geneseeq Technology Inc., Nanjing, P.R. China.; 4 Department of Pathology, Cancer Hospital Affiliated to Shanxi Medical University, Shanxi Province Cancer Hospital, Shanxi Hospital Affiliated to Cancer Hospital, Chinese Academy of Medical Sciences, Taiyuan, P.R. China.

## Abstract

Mucosal melanoma exhibits limited responsiveness to anti-PD-1 therapy. However, a subgroup of mucosal melanomas, particularly those situated at specific anatomic sites like primary malignant melanoma of the esophagus (PMME), display remarkable sensitivity to anti-PD-1 treatment. The underlying mechanisms driving this superior response and the DNA methylation patterns in mucosal melanoma have not been thoroughly investigated. We collected tumor samples from 50 patients with mucosal melanoma, including 31 PMME and 19 non-esophageal mucosal melanoma (NEMM). Targeted bisulfite sequencing was conducted to characterize the DNA methylation landscape of mucosal melanoma and explore the epigenetic profiling differences between PMME and NEMM. Bulk RNA sequencing and multiplex immunofluorescence staining were performed to confirm the impact of methylation on gene expression and immune microenvironment. Our analysis revealed distinct epigenetic signatures that distinguish mucosal melanomas of different origins. Notably, PMME exhibited distinct epigenetic profiling characterized by a global hypermethylation alteration compared with NEMM. The prognostic model based on the methylation scores of a 7-DMR panel could effectively predict the overall survival of patients with PMME and potentially serve as a prognostic factor. PMME displayed a substantial enrichment of immune-activating cells in contrast to NEMM. Furthermore, we observed hypermethylation of the *TERT* promoter in PMME, which correlated with heightened CD8^+^ T-cell infiltration, and patients with hypermethylated *TERT* were likely to have improved responses to immunotherapy. Our results indicated that PMME shows a distinct methylation landscape compared with NEMM, and the epigenetic status of *TERT* might be used to estimate prognosis and direct anti-PD-1 treatment for mucosal melanoma.

**Significance:**

This study investigated the intricate epigenetic factor of mucosal melanomas contributed to the differential immune checkpoint inhibitor response, and found that PMME exhibited a global hypermethylation pattern and lower gene expression in comparison to NEMM. *TERT* hypermethylation may contribute to the favorable responses observed in patients with mucosal melanoma undergoing immunotherapy.

## Introduction

Mucosal melanoma, an infrequent yet formidable melanoma subtype, originates from melanocytes residing within mucosal surfaces found throughout the body, including the oral cavity, gastrointestinal tract, genitourinary tract, and various anatomic regions (1). In contrast to cutaneous melanoma, mucosal melanoma has traditionally received less attention in the landscape of melanocytic malignancies. However, its unique clinical characteristics, marked aggressiveness, and varying responses to therapy have progressively drawn scientific interest (2).

While immune checkpoint inhibitors (ICI) have exhibited remarkable potential in the treatment of cutaneous melanoma, the majority of patients with mucosal melanoma display resistance to these therapies, and the precise underlying mechanisms responsible for this resistance remain incompletely elucidated within the context of mucosal melanoma (3–6). It is noteworthy that mucosal melanomas frequently exhibit a lower tumor mutational burden (TMB) compared with their cutaneous counterparts (7). This reduced TMB potentially translates to fewer neoantigens, thereby potentially compromising the effectiveness of ICIs. Furthermore, the identification of reliable biomarkers for predicting ICI responses in mucosal melanoma remains a pressing challenge, introducing a degree of imprecision in patient selection for such treatments.

Our investigations have unveiled the profound heterogeneity inherent in mucosal melanoma, with response variations contingent on the specific anatomic site. Within this intricate landscape, certain subtypes of mucosal melanoma exhibit superior responses to ICIs when compared with others. Notably, primary malignant melanoma of the esophagus (PMME), despite generally conferring a more adverse prognosis than non-esophageal mucosal melanoma (NEMM), paradoxically presents significantly heightened responsiveness to anti-PD-1 immunotherapy (8–10). The imperative to delineate the mechanistic underpinnings of this differential immunotherapeutic susceptibility is underscored by its potential to inform patient selection for ICI treatments. To elucidate the specific mechanisms underlying the more favorable immunotherapy responses observed in PMME compared with NEMM can be conducive to select patients with mucosal melanoma who may benefit from ICIs.

Genomic sequencing investigations have revealed an elevated prevalence of mutations in classical cutaneous driver genes among PMME cases when contrasted with NEMM instances (10, 11). In addition, transcriptomic analyses have indicated that PMME frequently exhibits an inclination toward heightened proliferation and inflammatory features (10). However, epigenetic elements, encompassing DNA methylation patterns, histone modifications, and chromatin remodeling, which constitute pivotal determinants of tumor dynamics, have hitherto not been adequately explored, particularly in relation to ICI responsiveness, within the domain of mucosal melanoma. Moreover, the DNA methylation landscape specific to PMME has remained an uncharted territory, offering an untapped reservoir of insight into the role of epigenetic factors underpinning the enhanced response of PMME to PD-1 therapy.

In this study, we undertake the meticulous characterization of the DNA methylation landscape in mucosal melanoma. Our study encompasses a cohort of 50 mucosal melanoma cases, including 31 PMME and 19 NEMM cases, with the aim of elucidating the heterogeneity within mucosal melanoma, unraveling the potential of specific gene epigenetic status as a prognostic biomarker and for the precise selection of mucosal melanoma patients who stand to derive maximal benefit from anti-PD-1 immunotherapy. In addition, we delve into the intricate interplay between epigenetic modifications, gene expression profiles, and the multifaceted tumor microenvironment, thereby offering a comprehensive vista of the clinical implications stemming from our findings.

## Materials and Methods

### Patients and Samples

In this retrospective study, 50 patients with mucosal melanoma with available samples were included, including 31 patients with PMME and 19 patients with NEMM. The patients were diagnosed and treated at three medical centers in China, namely Beijing Cancer Hospital, Cancer Hospital Chinese Academy of Medical Sciences, and Shanxi Provincial Cancer Hospital, over the period from 2012 to 2022 (last follow-up in January 2023). Tumor and paracancerous tissue samples were obtained from resection or biopsy procedures and were preserved in formalin-fixed paraffin-embedded (FFPE) tissue blocks. All the paracancerous tissues were distal tissues of tumors. Each of these tissues underwent thorough examination, including hematoxylin and eosin staining, and was verified by a pathologist to be normal, with the absence of melanoma tumor cells. Clinical information, including patient age, sex, and primary site, was collected from the clinical archives of the participating hospitals. Progression-free survival (PFS) was defined as the time from the start of treatment to progression or last follow-up. Overall survival (OS) was defined as the time from diagnosis to death or last follow-up. This study was approved by the Institutional Review Board of the aforementioned hospitals, and all patients signed informed consent forms prior to sample collection.

### Targeted Bisulfite Sequencing

The DNA was first extracted from the FFPE samples, then treated with bisulfite to construct the sequencing library. Targeted bisulfite sequencing was performed using SeqCap Epi 4M CpGiant probes (Roche) on the Illumina Hiseq platform (Illumina). Raw sequencing data were first demultiplexed by bcl2fastq and then trimmed by Trimmomatic as part of the quality control (QC) protocol (12). The qualified reads were then mapped onto the human reference genome (GRCh37/UCSC hg19) using the bisulfite sequence aligner Bismark after PCR duplicates removal by deduplicate_bismark (13).

### Identification of Differentially Methylated Region

The methylKit package (version 1.12.0) was used to identify differentially methylated regions (DMR) in R (version 4.2.1; ref. 14). CpG clusters were tiled into 100 bp windows with 100 bp step after filtering out low coverage (<5X) to ensure better detection of DMRs based on parameters that were previously reported by Ziller and colleagues (15). The methylation level of each DMR was calculated using the total methylated cytosines divided by the total CpGs within each window. Logistic regression was applied to calculate the methylation difference as well as the FDR between different groups (i.e., PMME vs. normal or PMME vs. NEMM). The threshold for DMR identification was *q* < 0.01 and |methylation difference| > 0.2.

### RNA Extraction and Sequencing

Total RNA from FFPE samples was extracted using miRNeasy FFPE kit (QIAGEN). Ribosomal RNA was depleted using KAPA Stranded RNA sequencing (RNA-seq) Kit with RiboErase (HMR; KAPA Biosystems). Library preparations were performed with KAPA Stranded RNA-seq Library Preparation Kit (Roche). Library concentration was determined by KAPA Library Quantification Kit (KAPA Biosystems), and library quality was accessed by Agilent High Sensitivity DNA kit on Bioanalyzer 2100 (Agilent Technologies), which was then sequenced using the DNBSEQ-T7RS Sequencing System (BGI) per the manufacturer's guidelines.

QC was performed with Trimmomatic (version 0.33). STAR (version 2.7.3a) is used for transcriptome mapping followed by isoform and gene-level quantification performed by RSEM (version 1.3.0). Differential expression analysis was conducted by R packages DESeq2 (version 1.16.1). Differentially expressed (DE) genes were selected by fold change > 2 and *P* value < 0.05.

### Multiplex Immunofluorescence Staining and Analysis

FFPE sections, 4-µm thick, were subjected to staining using an automated staining system (BOND-MAX; Leica Microsystems) with the 6-color Kit (Abcarta) following the manufacturer's instructions. The markers were grouped into two distinct panels: a macrophage panel and a T-cell panel. The macrophage panel included SOX10 (EP268, ZSGB-BIO), CD68 (KP1, MXB Biotechnologies), CD163 (10D6, ZSGB-BIO), CD80 (ab134120, Abcam), and CD206 (ab64693, Abcam). T-cell panel consisted of SOX10, CD4 (458G4A1, Abcarta), CD8 (815R4B2, Abcarta), CD69 (ab233396, Abcam), and CD45RO (UCHL-1, MXB Biotechnologies). Normal human tonsil FFPE tissues were also stained with and without primary antibodies to serve as positive and negative controls, respectively. All stained slides were scanned using a digital pathology slide scanner (× 40, KF-PRO-005, KFBIO). Trained pathologists utilized image analysis software (HALO 3.3, Indica Labs) to discern and quantify cell phenotypes. Moreover, each case was subject to manual review to ensure consistency and accuracy. The positive rate of immune cells is defined as the proportion of immune cells to total cells. In addition, the proportion of M1 or M2 macrophages to the total number of macrophages is used for comparison between PMME and NEMM.

### Statistical Analysis

Kaplan–Meier analysis was used to estimate the PFS and OS, and the statistical difference was compared using the log‐rank test. Univariate and multivariate COX analyses were conducted to evaluate the impact of DMR on OS in patients with PMME. The COX model was constructed using DMRs identified from statistically significant results in multivariate analysis, and its performance was tested using leave-one-out cross-validation (LOOCV). The immune microenvironment was analyzed using the CIBERSORT-ABS approach (16). The Kruskal–Wallis test and Wilcoxon rank-sum test were conducted to compare different groups, and multiple comparisons were adjusted using the Benjamini–Hochberg approach. The Pearson correlation coefficient was calculated to assess the correlation between transcriptome and epigenome data. Gene set enrichment analysis (GSEA) was performed using the ClusterProfiler (version 4.6.2) R package. A two-sided *P* values of less than 0.05 was considered statistically significant. All statistical analyses were performed R software (version 4.2.1).

### Data Availability Statement

The raw sequence data generated in this study have been deposited in the Genome Sequence Archive (GSA-Human: https://ngdc.cncb.ac.cn/gsa-human) under the accession number HRA006934. Because of patient privacy requirements, access to these data is restricted and can be made granted upon a reasonable request to the corresponding author.

### Ethics Approval and Consent to Participate

This study has been conducted in compliance with local Institutional Review Board policies, and all patients signed informed consent forms prior to sample collection and consented to the publication of related clinical information and data.

## Results

### Patient Characteristics

A total of 50 patients with mucosal melanoma were included in this study, including 31 patients with PMME and 19 patients with NEMM (9 nasal/oral, 6 genital, and 4 anorectal). The basic patient characteristics are listed in Table 1. All patients had resected or biopsy tumor tissue samples available and 9 patients with PMME had matched paracancerous tissue samples to be used as normal controls. The PMME group predominantly consisted of male patients, with 28 out of 31 cases (90.3%) being male. In contrast, the NEMM group had a relatively equal distribution of male (9/19, 47.4%) and female (10/19, 52.6%) patients. The median age of patients with PMME was 55 years (range, 38–74), while for patients with NEMM it was 57 years (range, 40–76). The majority of patients with PMME (29/31, 93.5%) received surgical resection, whereas 52.6% (10/19) of patients with NEMM received surgical resection.

**TABLE 1:** Demographic and clinical characteristics of the patients with PMME and NEMM included in the study

Characteristic	PMME (*n* = 31)	NEMM (*n* = 19)
Sex		
Female	3 (9.7%)	10 (52.6%)
Male	28 (90.3%)	9 (47.4%)
Age, years	55 (38–74)	57 (40–76)
<55	15 (48.4%)	9 (47.4%)
≥55	16 (51.6%)	10 (52.6%)
Primary site		
Esophagus	31 (100%)	0 (0%)
Nasal/oral	0 (0%)	9 (47.4%)
Genital	0 (0%)	6 (31.6%)
Anorectal	0 (0%)	4 (21.1%)
Metastases		
M0	17 (54.8%)	12 (63.2%)
M1	14 (45.2%)	7 (36.8%)
Surgical treatment		
Yes	29 (93.5%)	10 (52.6%)
No	2 (6.5%)	9 (47.4%)
Systemic immunotherapy		
Yes	3 (9.7%)	19 (100%)
No	28 (90.3%)	0 (0%)

### PMME Exhibits A Higher Frequency of Gene Promoter Hypermethylation

**FIGURE 1:**
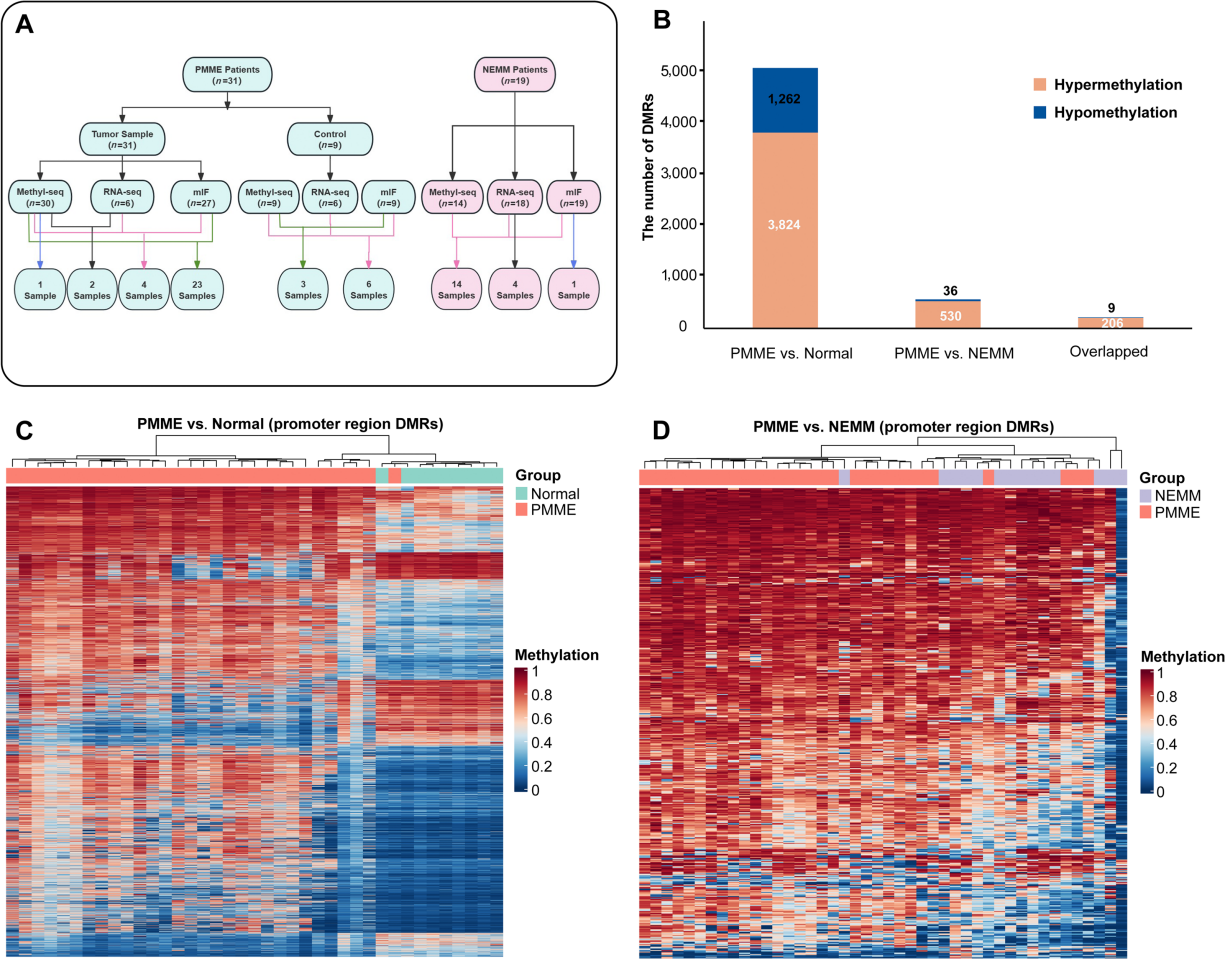
PMME had a distinct epigenetic profile when compared with NEMM and normal controls. **A,** Schematic representation of the study design and methodology. Methyl-seq, methylation sequencing; mIF, multiplex immunofluorescence; QC, quality control. **B,** The number of hypermethylated and hypomethylated DMRs between PMME, NEMM, and normal controls. **C,** The unsupervised clustering of DMRs in the promoter regions between PMME and normal controls. **D,** The unsupervised clustering of DMRs in the promoter regions between PMME and NEMM.

All tissue samples underwent targeted bisulfite sequencing, and the results of 30 PMME samples, nine paracancerous normal esophageal tissue samples, and 14 NEMM samples passed the QC for DNA methylation analyses (Fig. 1A). The comparison between PMME and paracancerous normal esophageal tissue samples revealed a total of 5,086 DMRs in the promoter region. Among these DMRs, 3,824 (75.2%) were found to be hypermethylated, while 1,262 (24.8%) were hypomethylated in PMME (Fig. 1B), indicating a global hypermethylation alteration in PMME compared with normal samples. The unsupervised clustering using these DMRs could clearly distinguish PMME from normal samples (Fig. 1C). In the comparison between PMME and NEMM, a total of 566 promoter DMRs were identified, and the majority (530/566, 93.6%) of these DMRs were hypermethylated in PMME (Fig. 1B). The unsupervised hierarchical cluster analysis based on these DMRs could separate 86.7% (26/30) of the PMME samples from NEMM samples (Fig. 1D). We further analyzed the overlapping DMRs between “PMME vs. Normal” and “PMME vs. NEMM,” 215 promotor DMRs were identified. Among these DMRs, 206 (95.8%) regions exhibited hypermethylation, while only nine regions showed hypomethylation specifically in PMME compared with both normal esophageal tissue samples and NEMM (Fig. 1B). These findings suggest that during the progression of PMME, the tumors acquire a hypermethylation phenotype, distinguishing them from NEMM.

In addition to epigenetic analysis, RNA-seq was conducted on 10 PMME samples, 19 NEMM samples, and six normal samples to investigate the gene expression profile. After QC, six PMME samples, 18 NEMM samples, and six normal samples were included for further analysis (Fig. 1A). In particular, we identified 8,602 DE genes when comparing PMME and normal samples, and 3,088 DE genes were found when comparing PMME with NEMM. Furthermore, in line with the higher DNA methylation levels observed in PMME, a larger number of genes were downregulated in PMME compared with normal samples or NEMM (Supplementary Fig. S1A–S1D). These findings suggest that PMME displays unique epigenetic characteristics, characterized by distinct hypermethylation patterns, as well as with significant variations in the transcriptome. These differences in epigenetic and transcriptomic profiles may contribute to the distinctive clinical features observed in PMME.

### Epigenetic-based Models for Prognosis Estimation in PMME

**FIGURE 2:**
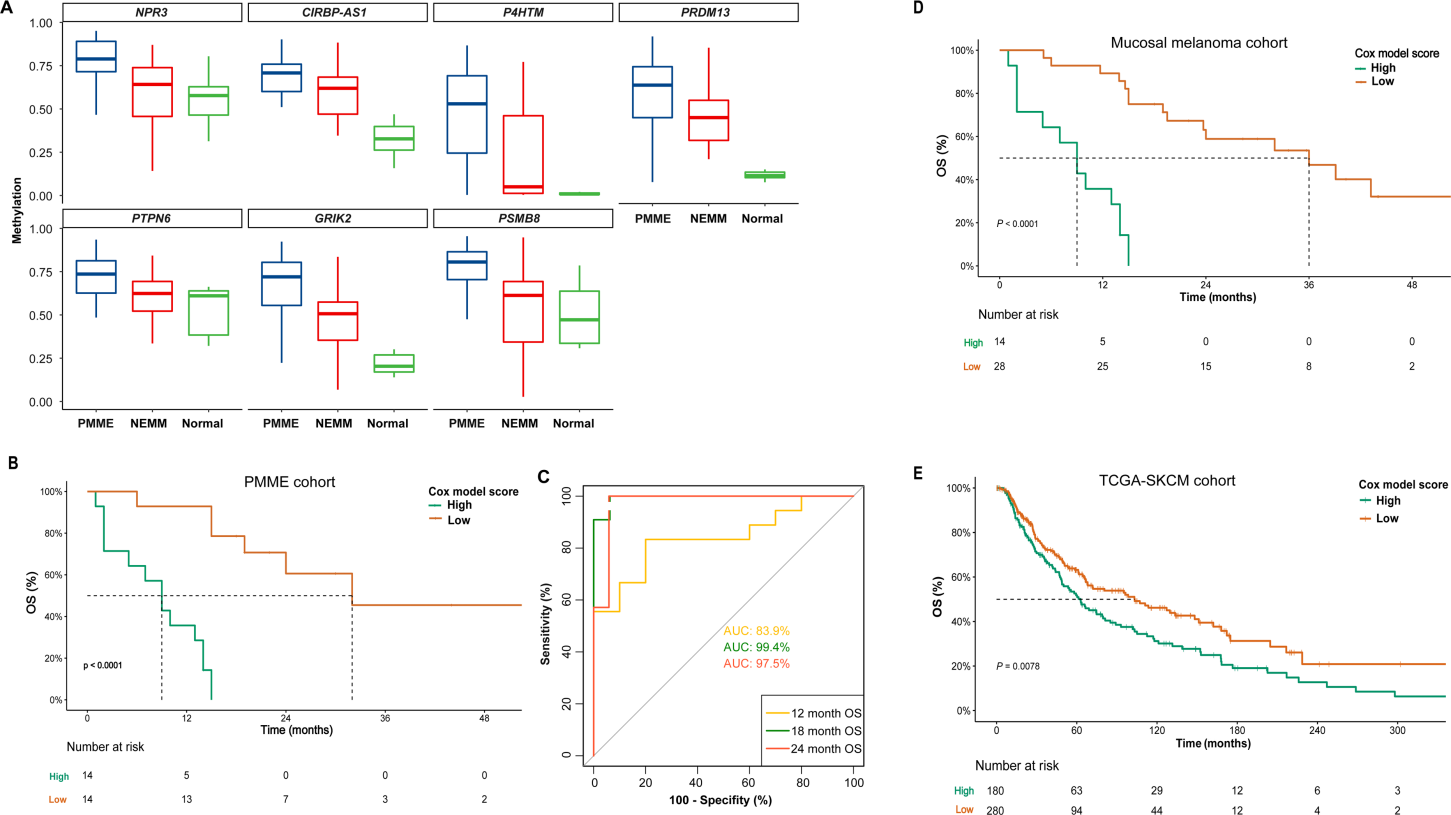
Association of epigenetic features with prognosis in patients with PMME. **A,** Comparison of methylation levels of selected DMRs (statistically significant in multivariate analysis) among PMME, NEMM, and normal controls. **B,** Kaplan–Meier curve of OS of patients with PMME stratified on the basis of the risk score derived from the 7-DMR panel–based COX model. **C,** ROC curve depicting the prediction performance of 7-DMR panel–based COX model for patients survival status at 12, 18, and 24 months, assessed using LOOCV. Kaplan–Meier curve of OS in the entire queue of patients with mucosal melanomas (**D**) and inTCGA-SKCM cohort stratified on the basis of the risk score derived from the 7-DMR panel–based COX model (**E**).

As 215 promoter DMRs were found to be distinguishably methylated in PMME, we tried to investigate whether some of these epigenetic features could serve as prognostic markers for PMME. We merged the original 215 DMRs (i.e., 100 bp each) into 145 DMRs by combining adjacent DMRs. Thirty DMRs were found to be significantly associated with OS using univariate COX regression (Supplementary Table S1). By performing multivariate COX analysis, it was determined that seven DMRs namely *NPR3*, *CIRBP-AS1*, *P4HTM*, *PRDM13*, *PTPN6*, *GRIK2*, and *PSMB8*, remained to be statistically significant (Supplementary Table S1; Supplementary Fig. S2). These DMRs were found to be hypermethylated in PMME than NEMM and normal samples (Fig. 2A). This suggests that hypermethylation of these specific genes could potentially serve as independent prognostic factors for PMME. To evaluate the prognostic value of these DMRs, a Cox model was established using the seven DMRs. The results showed that patients with PMME with higher scores based on the 7-DMR panel had significantly worse OS (*P* < 0.001, Fig. 2B). To further validate the predictive capability of the model, LOOCV was performed. The LOOCV results indicated that the model effectively predicted the OS of patients with PMME at 12, 18, and 24 months after diagnosis, with AUC being 83.9%, 99.4%, and 97.5%, respectively (Fig. 2C). This suggests that these specific epigenetic features represented by the identified 7-DMR panel have the potential to serve as biomarkers for predicting the prognosis of PMME. The reliability of the 7-DMR panel as a prognostic factor was also confirmed in the entire queue of mucosal melanomas, encompassing PMME and NEMM (Fig. 2D).

We further undertook an analysis of the association of the 7-DMR score with survival in The Cancer Genome Atlas (TCGA)-skin cutaneous melanoma (SKCM) cohort. TCGA-SKCM methylation array data for 475 cases were obtained from the Genomic Data Commons. After excluding 15 cases with missing OS information and two adjacent normal samples, a final set of 458 TCGA-SKCM samples were included in the analysis. We identified CG sites covered by DMRs in this study and calculated the beta mean values to model the methylation of seven DMRs. The results demonstrated that the 7-DMR panel showed predictive value for survival in the broader context of SKCM (Fig. 2E). These findings suggest that the 7-DMR panel could potentially serve as a prognostic factor for melanoma across different subtypes.

### Enrichment of Various Immune Cells and Immune-related Pathways in PMME

**FIGURE 3:**
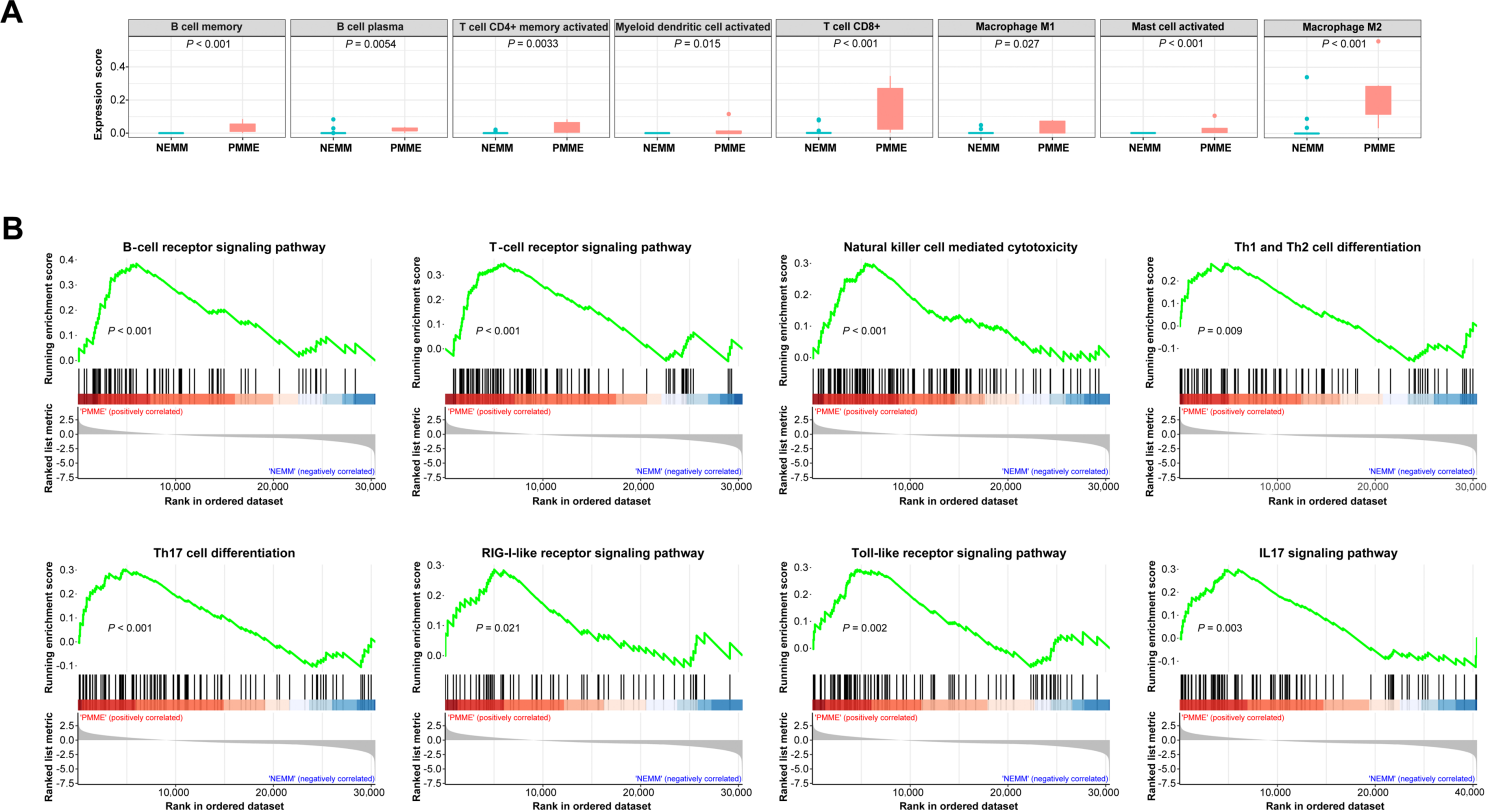
Increased immune cell infiltration and enriched immune-related pathways in PMME compared with NEMM. **A,** Comparison of infiltrated immune cells between PMME and NEMM. **B,** Significantly enriched immune-related pathways identified through GSEA when comparing PMME and NEMM.

Considering that PMME demonstrated better responses to immunotherapy than NEMM, the tumor immune microenvironment was investigated using the transcriptomic data to understand the differences between PMME and NEMM. CIBERSORT-ABS algorithm was used to estimate the immune cell infiltration differences between the two cohorts. As shown in Fig. 3A, PMME tumors showed significant enrichment of various immune cells, including proinflammatory immune cells, such as M1 macrophages, plasma B cells, activated CD4^+^ memory T cells, and CD8^+^ T cells, indicating a more pronounced immune response in PMME. In addition, a few anti-inflammatory immune cells, particularly M2 macrophage, were also enriched in PMME tumors. Furthermore, GSEA revealed 13 immune-related pathways were significantly enriched in PMME (Fig. 3B; Supplementary Table S2), including antigen processing and presentation, B-cell receptor signaling, T-cell receptor signaling, NOD-like receptor signaling, natural killer cell–mediated cytotoxicity, and IL17 signaling pathway, etc. The enrichment of these pathways suggests that PMME is more likely to be associated with a higher number of immune-related processes compared with NEMM.

### Correlation Between Epigenetic Features and Immune Cell Infiltration in PMME

**FIGURE 4:**
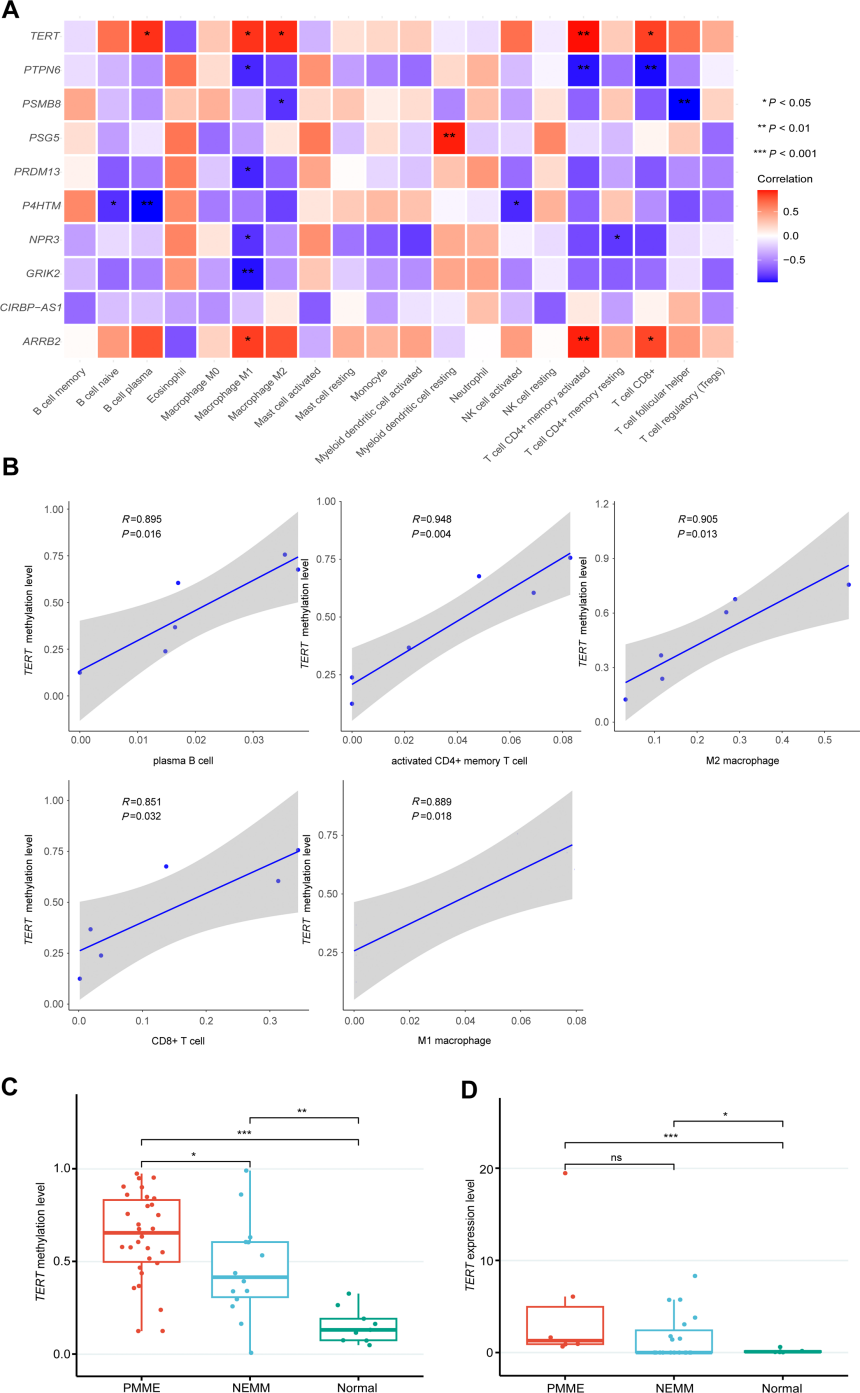
Role of *TERT* hypermethylation in PMME and its impact on immunologic signatures. **A,** Heat map depicting the association between hypermethylated genes and the immune cell infiltration in PMME. **B,** Linear regression analysis demonstrating the correlation between *TERT* methylation and immune infiltration in PMME. **C,** Comparison of *TERT* methylation levels among PMME, NEMM, and controls (*, *P* < 0.05; **, *P* < 0.01; ***, *P* < 0.001). **D,** Comparison of *TERT* expression levels among PMME tumors, NEMM, and controls.

We further investigated the relationship between DNA methylation profile of the 145 promotor DMRs and immune cell infiltration in patients with PMME. The majority of these hypermethylated genes displayed distinct associations with various immune cell types. Intriguingly, we observed that the methylation levels of *TERT* and *ARRB2* were positively linked with the infiltration of M1 macrophages, activated CD4^+^ memory T cells, and CD8^+^ T cells (Fig. 4A and B). In addition, the methylation of *TERT* exhibited a positive correlation with plasma B cells in PMME, which closely mirrors the differences in immune infiltration between PMME and NEMM. The association between the methylation levels of *TERT* and the 7-DMR panel with immune cell infiltration was also examined in patients with NEMM. However, a distinct pattern emerged, as no significant association was observed between the methylation level of *TERT* and immune cell infiltration in NEMM (Supplementary Fig. S3). Therefore, we compared the methylation level of *TERT* in PMME, NEMM, and normal controls, and the result revealed that the *TERT* methylation level in PMME was significantly higher than that of NEMM and normal controls (Fig. 4C). However, the expression levels of *TERT* were not different between PMME and NEMM, but both groups showed significantly higher expression levels compared to the normal controls (Fig. 4D). The distinct immune profile implies that the hypermethylation of *TRET* might be related to the elevated immune infiltration observed in PMME.

### More Immune-activating Cells Infiltrate in PMME Than in NEMM

**FIGURE 5:**
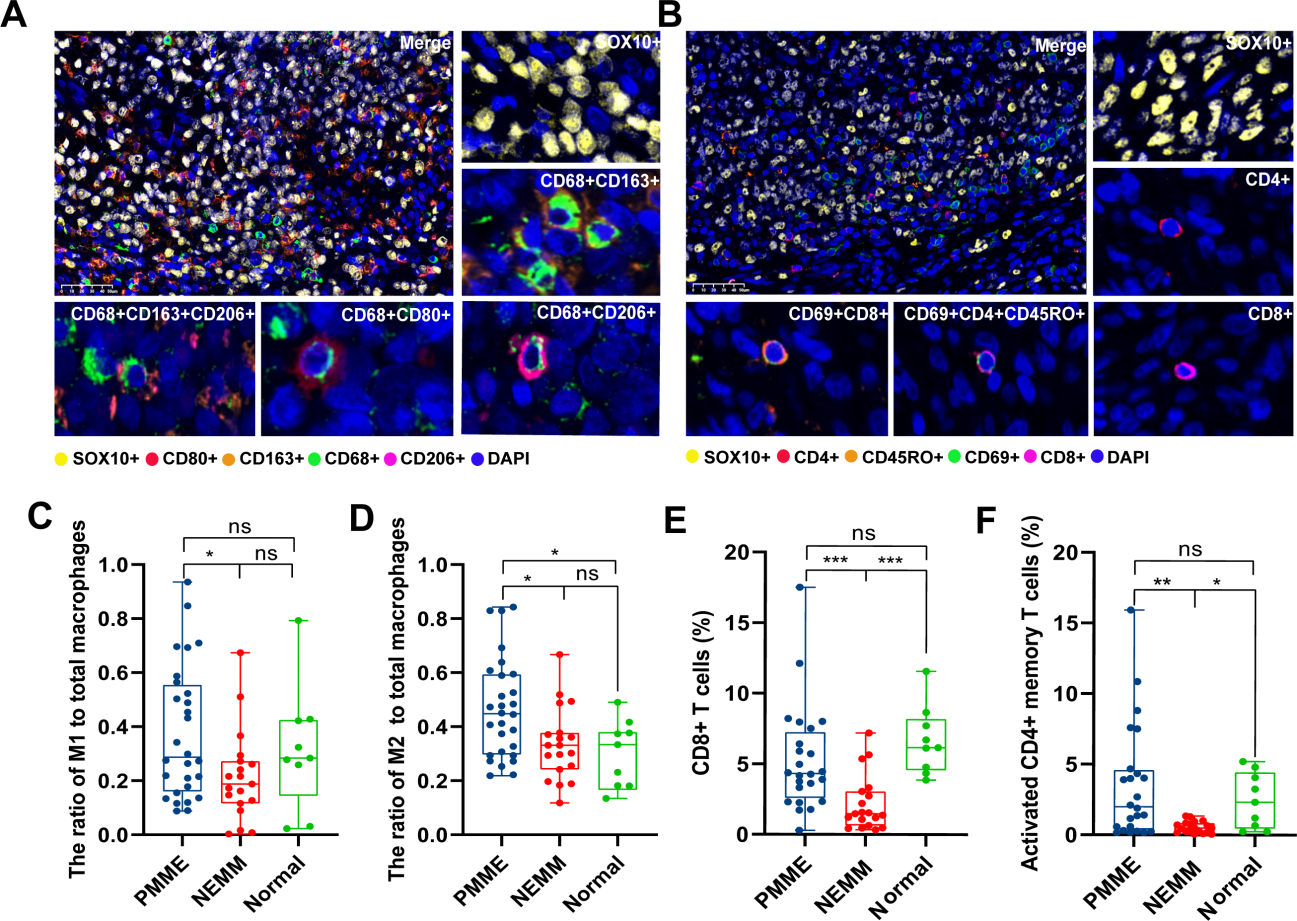
More M1 and M2 macrophages, CD8^+^ T cells, and activated CD4^+^ memory T cells infiltrated in PMME compared with NEMM. **A,** Representative mIF image of M1 and M2 macrophages. **B,** Representative mIF image of CD8^+^ T cells and activated CD4^+^ memory T cells. SOX10, CD68, CD45RO, and CD69 as markers for melanoma cells, macrophages, memory T, and activated T cells cells, respectively. CD80 was employed to label M1 macrophages, while CD163 and CD206 were used to label M2 macrophages. The ratio of M1 (**C**) and M2 (**D**) to total macrophages in PMME, NEMM, and controls. The positive rate of CD8^+^ T cells (**E**) and activated CD4^+^ memory T cells (**F**) in PMME, NEMM, and controls (*, *P* < 0.05; **, *P* < 0.01; ***, *P* < 0.001).

According to the transcriptome analysis, we observed that M1 and M2 macrophages, CD8^+^ T cells, and activated CD4^+^ memory T cells exhibited higher infiltration levels in PMME compared with NEMM. Moreover, the infiltration levels of these immune cells in PMME was found to be associated with the methylation level of *TERT*. Therefore, we conducted multiplex immunofluorescence (mIF) staining on 27 PMME tissues, 19 NEMM tissues, and nine normal samples to validate these findings. Four NEMM cases were excluded from the T-cell panel analysis due to QC failures. M1 macrophages were identified as cells coexpressing CD68 and CD80, while M2 macrophages were identified as cells coexpressing CD68 with CD163 or CD206 (Fig. 5A), and activated CD4^+^ memory T cells were identified by coexpressing CD4 with CD45RO and CD69 (Fig. 5B). Quantitative analysis demonstrated a significantly higher proportion of M1 macrophages in PMME compared with NEMM (Fig. 5C), along with a higher ratio of M2 macrophages in PMME relative to both NEMM and normal samples (Fig. 5D), and the M1/M2 ratio also tended to higher in PMME compared with NEMM. In the context of T cells, the results revealed that the infiltration levels of CD8^+^ T cells and activated CD4^+^ memory T cells were comparable between PMME and normal samples, but both were significantly higher than those observed in NEMM samples (Fig. 5E and F). These findings validate that the enhanced infiltration of CD8^+^ T cells and activated CD4^+^ T cells within the tumor microenvironment of PMME. Moreover, the macrophages present in PMME displayed a proinflammatory phenotype, signifying a potentially heightened immune-activating profile in PMME relative to NEMM.

### *TERT* Methylation Correlates with the Enrichment of Immune-related Pathways and Response to Immunotherapy

**FIGURE 6:**
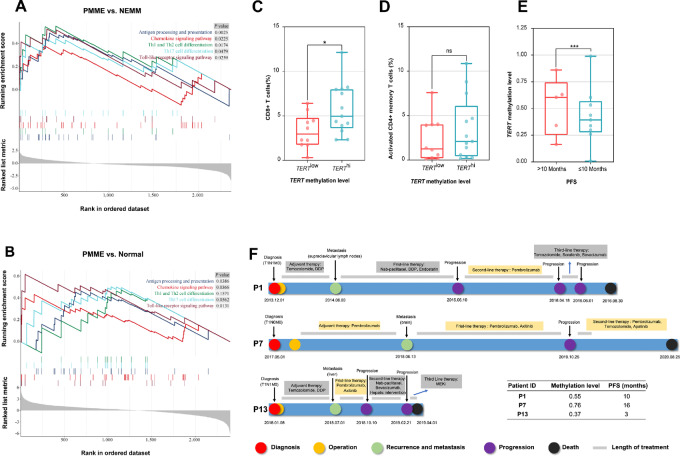
Association between *TERT* hypermethylation and immunotherapy responses. **A,** Significant immune-related pathways that were derived from GSEA of *TERT* methylation-related genes when comparing PMME and NEMM. **B,** Significant immune-related pathways derived from GSEA of *TERT* methylation-related genes when comparing PMME and normal controls. The infiltration level differences of CD8^+^ T cells (**C**) and activated CD4^+^ memory T cells (**D**) in patients with *TERT*^hi^ and *TERT*^low^ PMME (*, *P* < 0.05). **E,** PFS differences between patients with *TERT*^hi^ and *TERT*^low^ NEMM receiving immunotherapy. **F,** Case demonstrations of 3 patients with PMME (P1, P7, and P13) who received anti-PD-1 immunotherapy during their treatment course. DDP, cisplatin.

We then performed GSEAs using gene expressions that were associated with *TERT* methylation. By comparing PMME and NEMM samples, these *TERT*-associated genes led to significant enrichments of multiple immune-related pathways, including antigen processing and presentation, chemokine signaling, Th1 and Th2 cell differentiation, Th17 cell differentiation, and Toll-like receptor signaling (Fig. 6A). Similar results were obtained when compared between PMME and normal controls (Fig. 6B). We further divided patients with PMME into high and low *TERT* methylation groups using the median *TERT* methylation level of tumor and normal samples from the PMME cohort. The results showed that a total of 19 immune-related pathways were significantly enriched in the *TERT*^hi^ group (Supplementary Table S3). We further analyzed the correlation between the infiltration status of CD8^+^ T cells and activated CD4^+^ memory T cells with the methylation level of the *TERT* within PMME cohort using mIF staining. Consistent with the transcriptome analysis, the *TERT*^hi^ group exhibited a significantly higher infiltration of CD8^+^ T cells compared with the *TERT*^low^ group (Fig. 6C). However, there was no statistically significant difference observed in the infiltration level of activated CD4^+^ memory T cells between these two groups (Fig. 6D).

The association between *TERT* methylation and clinical outcomes were further analyzed. Patients with NEMM with better clinical responses to immunotherapy had significantly higher *TERT* methylation levels (Fig. 6E). Three patients with PMME (P1, P7, and P13) in our cohort received immunotherapy, and the immunotherapy response appeared to be related to *TERT* methylation levels (Fig. 6F). Notably, patients P1 and P7 exhibited higher *TERT* methylation levels (0.55 and 0.76, respectively) than patient P13 (0.37). After experiencing disease progression on first-line treatment of chemotherapy in combination with angiogenesis inhibitor, patient P1 received second-line pembrolizumab and achieved a PFS of 10 months. Similarly, patient P7 showed a promising response to immunotherapy in combination with angiogenesis inhibitor, with a PFS of 16 months for first-line pembrolizumab plus axitinib. In contrast, patient P13, who had hypomethylated *TERT*, received the same first-line treatment as patient P7, but only had a PFS of 3 months. These results suggest that *TERT* hypermethylation may be associated with improved responses to immunotherapy in mucosal melanoma.

## Discussion

The resistance of mucosal melanoma to anti-PD-1 mAbs has limited their utility in a subset of patients. However, PMME has demonstrated sensitivity to anti-PD-1 monotherapy, serving as a model to investigate and identify specific characteristics in this responsive patient population. Therefore, we navigate the intricate epigenetic terrain of mucosal melanomas to unravel the enigmatic factors contributing to the differential ICI response. In this study, we observed significant differences in the epigenetic and transcriptomic profiles between PMME, NEMM, and normal controls. Specifically, PMME exhibited a higher frequency of DNA methylations and lower levels of gene expression. By analyzing the 145 overlapping DMRs between “PMME vs. Normal” and “PMME vs. NEMM,” we constructed a 7-DMR panel to predict the prognosis of patients with PMME. In addition, we found that PMME was “hotter” than NEMM with increased immune cell infiltration and significant enrichment of immune-related pathways. Notably, these elevated immunologic signatures were more pronounced in the high *TERT* methylation group than in the low *TERT* methylation group, and patients with hypermethylated *TERT* displayed higher CD8^+^ T-cell infiltration and showed a favorable clinical response to immunotherapy in both PMME and NEMM, indicating a potential association between *TERT* methylation status and improved immunotherapy treatment outcomes. Overall, our findings provide potential prognostic biomarkers for PMME and contribute to the understanding of molecular and microenvironmental mechanisms underlying unique therapeutic responses in these patients.

Previous studies have consistently reported a higher incidence of PMME in male patients (9, 17), and our cohort showed similar results, with 90.3% of patients with PMME being males. Given the extremely poor prognosis of patients with PMME, it is crucial to develop methods for estimating the prognosis of these patients. As expected, patients with PMME with advanced stages had worse OS compared with those with early stages (18, 19). In addition, several studies have demonstrated that patients with PMME with positive lymph node metastasis have a poorer prognosis (9, 18, 20). However, the prognostic value of epigenetic markers in PMME remains unexplored. In our study, we identified the methylation status of the promoter region for multiple genes, including *NPR3*, *CIRBP*-*AS1*, *P4HTM*, *PRDM13*, *PTPN6*, *GRIK2*, and *PSMB8*, as potential independent biomarkers for the prognosis of PMME. Shang and colleagues reported the hypermethylation and downregulation of *CIRBP* were associated with poor prognosis in gynecologic cancers (21). Similarly, DiDonna and colleagues found that lower expression of *P4HTM* was indicative of worse patient survival (22). Consistent with these findings, we observed that the hypermethylation of *CIRBP* and *P4HTM* contributed to higher COX model scores and shorter OS. On the basis of these identified differentially methylated genes, we established the 7-DMR panel that could effectively predict the prognosis of this rare disease. These findings suggest that epigenetic features could serve as promising prognostic biomarkers for PMME. We also found that the 7-DMR panel not only effectively predicted the survival of mucosal melanoma patients but also showed predictive value for survival in cutaneous melanoma. These findings suggest that the 7-DMR panel could potentially serve as a prognostic factor for melanoma across different subtypes. Further analysis and investigation are needed to fully understand the specific roles and implications of these genes in melanoma.

Because of limited clinical evidence, there is currently no consensus on an effective treatment for PMME. Temozolomide-based adjuvant chemotherapy has shown improved clinical outcomes in patients with stage II/III mucosal melanoma (23), but its effectiveness in advanced PMME has been suboptimal (8). In recent years, ICIs targeting PD-1 and PD-L1 have become effective strategies for the treatment of various types of cancer (24). ICIs showed unsatisfactory efficacy in patients with mucosal melanoma both as anti-PD-1 monotherapy and in combination with ipilimumab (3–6), which could be attributed to the low TMB and PD-L1 expression (7, 25, 26). However, our previous study has demonstrated promising sensitivity of PMME to ICIs, with no significant impact of TMB or PD-L1 expression on immunotherapy differences between PMME and NEMM (10). In addition to the differential genetic, proliferative, and inflammatory features of PMME, our previous study revealed higher infiltration of CD8^+^ T cells and activated dendritic cells in PMME compared with NEMM (10). In this study, according to the transcriptome analysis, we further observed higher infiltration of B cells, activated CD4^+^ memory T cells, M1 and M2 macrophages in PMME compared with NEMM. The more infiltration of CD8^+^ T cells, activated CD4^+^ memory T cells, M1 and M2 macrophages were confirmed by the mIF staining. Macrophage polarization results in two main subtypes: M1 (classically activated macrophages) and M2 (alternatively activated macrophages; ref. 27). M1 macrophages are proinflammatory, proficient in antigen presenting, and capable of activating type I T-cell response, thus aiding in the suppression and targeting of tumor cells (28). Conversely, M2 macrophages are anti-inflammatory, with low antigen-presenting capability, high immunosuppressive effects, and the ability to promote cell proliferation and angiogenesis (28). In addition, M2 macrophages may also contribute to the resistance of chemotherapy, radiotherapy, and antiangiogenic treatments (29–31). Numerous previous studies have reported a positive correlation between M1 macrophage, increased M1/M2 ratio, and better prognosis and longer survival in different types of cancers (32–35). In our study, we observed significantly higher infiltration of M2 macrophage in PMME compared with NEMM, which may partially explain the poorer prognosis in PMME compared with NEMM. On the other hand, ICIs have been reported to induce a transition from M2 to M1 macrophages, thereby enhancing antitumor immunity (36). This may help explain the better responses to immunotherapy in PMME. The enrichment of immune cells and immune-related pathways in PMME indicates a potentially heightened immune response in PMME tumors. This finding aligns with the observation that PMME has shown better responses to immunotherapy compared with NEMM. The presence of infiltrated immune cells and the activation of immune-related pathways suggest a favorable tumor immune microenvironment in PMME, which may contribute to improved antitumor immune responses and treatment outcomes.

We found that the immune-infiltrating cells and immune-related signaling were elevated in PMME with high *TERT* methylation than those with low *TERT* methylation, similar to the differences observed between PMME and NEMM. In addition, a higher *TERT* methylation level was associated with a better response to immunotherapy. TERT is widely overexpressed in various cancers, including different subtypes of melanoma (37). Evidence suggests that TERT is a self-antigen with immunogenic properties. Telomerase-specific T-cell responses have been identified in patients with melanoma (37). TERT has the ability to expand cytotoxic CD8 T cells through TERT-derived peptides binding to MHC class I molecules (38). Moreover, TERT-derived peptides can bind to HLA class II molecules, stimulating CD4 Th1-cell responses (39). Preexisting anti-TERT Th1 responses have been associated with better response rates, longer PFS, and OS in patients with melanoma treated with ICIs (39). However, unlike most promoter methylations that are typically associated with gene silencing, *TERT* promoter hypermethylation has been reported to positively correlate with elevated *TERT* expression and telomerase activity in various cancers (37, 40). Lee and colleagues proposed an intriguing phenomenon wherein a *TERT* hypermethylated oncological region (THOR) located upstream of the *TERT* core promoter acts as a repressive element when unmethylated. Conversely, methylation of THOR counteracts this repressive effect (40). *TERT* methylation and expression have been reported to be closely related to the prognosis of patients with cancer, including mucosal melanoma (41, 42), although its effects on immune infiltration have been shown to be cancer type specific. Our results suggest that *TERT* methylation could potentially serve as a predictive biomarker for ICIs in mucosal melanoma. In our study, the *TERT* methylation level in PMME was significantly higher than that of NEMM and normal controls. Although both PMME and NEMM exhibited significantly higher *TERT* expression levels compared with the normal controls, the expression levels of *TERT* in PMME were not significantly higher than in NEMM. This observation might be attributed to the limited number of PMME samples that passed RNA-seq quality, and the methylation level of most of these PMME samples that underwent RNA-seq was comparable to NEMM. It is plausible that *TERT* promoter methylation might be an epiphenomenon of genome-wide hypermethylation in PMME. Therefore, the specific involvement of *TERT* methylation in the response to immunotherapy still requires further exploration in future studies.

Our study had several limitations that should be acknowledged. First, the number of patients with PMME who received immunotherapy in our cohort was limited, therefore, drawing definitive conclusions regarding the relationship between *TERT* methylation levels and the response to immunotherapy is challenging. Second, the rarity of mucosal melanoma poses a constraint on the size of our patient cohort and without an external mucosal melanoma validation group. Despite this limitation, the intriguing molecular findings from our study can serve as a valuable basis for future clinical investigations and meta-analyses aimed at gaining a better understanding of PMME and improving its treatment. Third, owing to factors like limited sample availability and the requirement for stringent sequencing QC, paracancerous tissue was not obtained from the NEMM cohort. In addition, not every PMME had paired normal control samples, and only a fraction of tumors had qualified transcriptomic data for subsequent analyses. These constraints may have influenced the comprehensiveness of our study and should be taken into consideration when interpreting the results.

In summary, our study involved comprehensive molecular analyses in patients with mucosal melanoma, leading to several key findings. First, we identified multiple epigenetic features that could be used as prognosis indicators for mucosal melanoma. Second, we observed distinct patterns of immune infiltration and immune-related pathways, particularly in the context of M2 and M1 macrophages, which could explain the poor OS but improved response to immunotherapy in PMME. In addition, we found that the level of *TERT* methylation could potentially serve as a predictive biomarker for immunotherapy in patients with mucosal melanoma. These findings contribute to decipher the enigmatic constituents contributing to differential ICI responsiveness, and furnish a foundation for personalized therapeutic modalities, prognostic indices, and biomarker identification germane to the multifarious domain of mucosal melanoma.

## Supplementary Material

Table S1 Supplementary Table 1. The univariate and multivariate COX analyses for OS associated DMRs in PMME patients.

Table S2 Supplementary Table 2. The immune-related pathways that were enriched in PMME when compared with NEMM using GSEA analysis.

Table S3 Supplementary Table 3. The immune-related pathways that were enriched in TERT methylation high group versus TERT methylation low group using GSEA analysis.

Figure S1 Supplementary Figure S1. PMME were generally associated with relatively lower levels of gene expression. (A) Vocanal plot and (B) unsupervised clustering of the top 50 differentially expressed (DE) genes between PMME and normal control samples. (C) Vocanal plot and (D) unsupervised clustering of the top 50 DE genes between PMME and NEMM.

Figure S2 Supplementary Figure S2. Diagram illustrating the genomic loci of the 7-DMR panel.

Figure S3 Supplementary Figure S3. Heatmap depicting the association between methylation status of TERT and genes within 7-DMR panel and the immune cell infiltration in NEMM.
