# Supplementary material for: Comparative Epigenetic Profiling Reveals Distinct Features of Mucosal Melanomas Associated with Immune Cell Infiltration and Their Clinical Implications
Source: Cancer Res Commun. 2024 May 28;4(5):1351–62. doi: 10.1158/2767-9764.CRC-23-0406 (PMC11131765; doi:10.1158/2767-9764.CRC-23-0406)
Supplement: Figure S1 — Supplementary Figure S1. PMME were generally associated with relatively lower levels of gene expression. (A) Vocanal plot and (B) unsupervised clustering of the top 50 differentially expressed (DE) genes between PMME and normal control samples. (C) Vocanal plot and (D) unsupervised clustering of the top 50 DE genes between PMME and NEMM. [file crc-23-0406-s04.pdf]

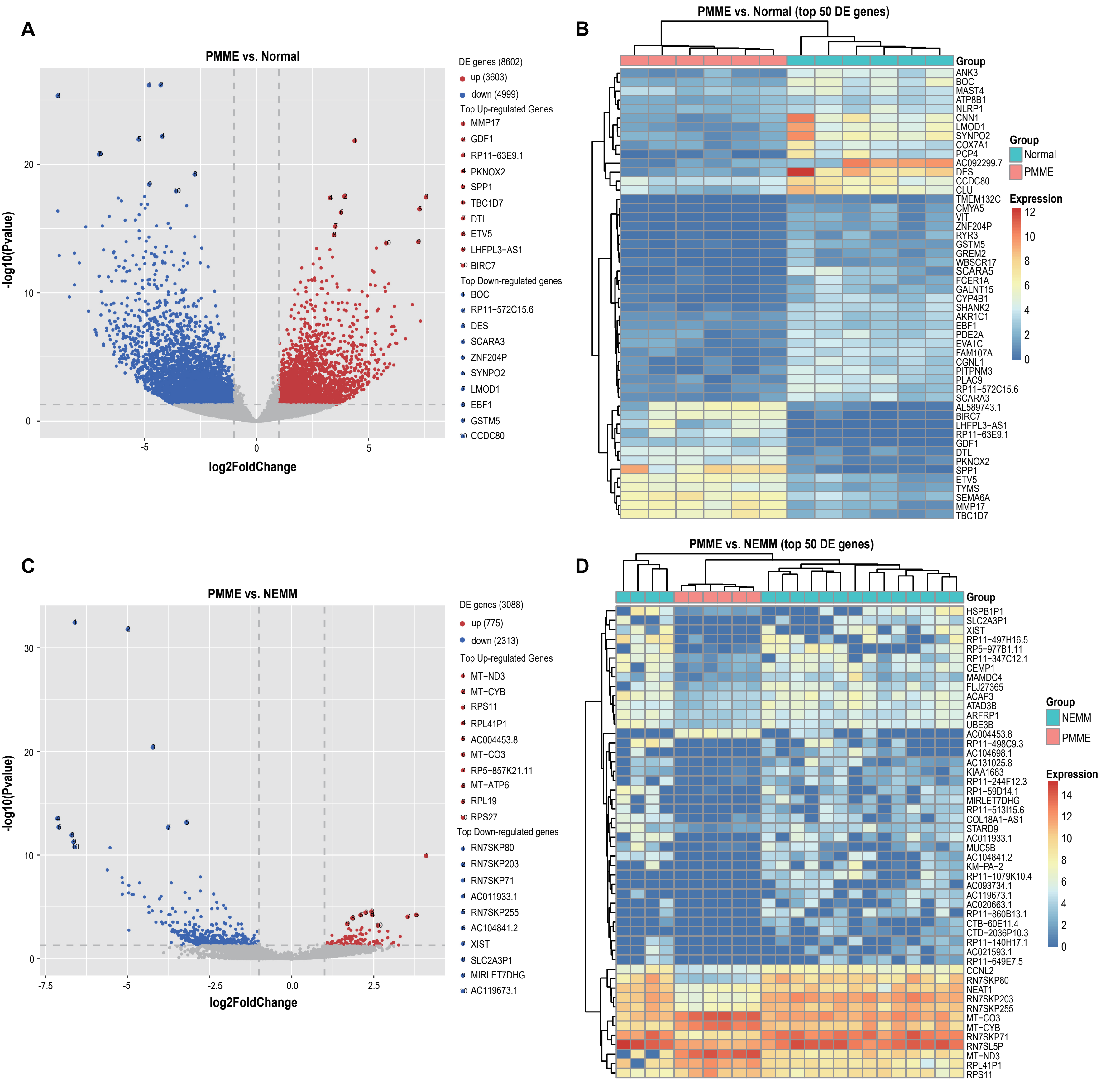

Supplementary Figure S1 .

PMME were generally associated with relatively lower levels of gene expression. (A) Vocanal plot and (B) unsupervised clustering of the top 50 differentially expressed (DE) genes between PMME and normal control samples. (C) Vocanal plot and (D) unsupervised clustering of the top 50 DE genes between PMME and NEMM.
