# Supplementary figures and images for: Comparative Epigenetic Profiling Reveals Distinct Features of Mucosal Melanomas Associated with Immune Cell Infiltration and Their Clinical Implications
Source: Cancer Res Commun. 2024 May 28;4(5):1351–62. doi: 10.1158/2767-9764.CRC-23-0406 (PMC11131765; doi:10.1158/2767-9764.CRC-23-0406)

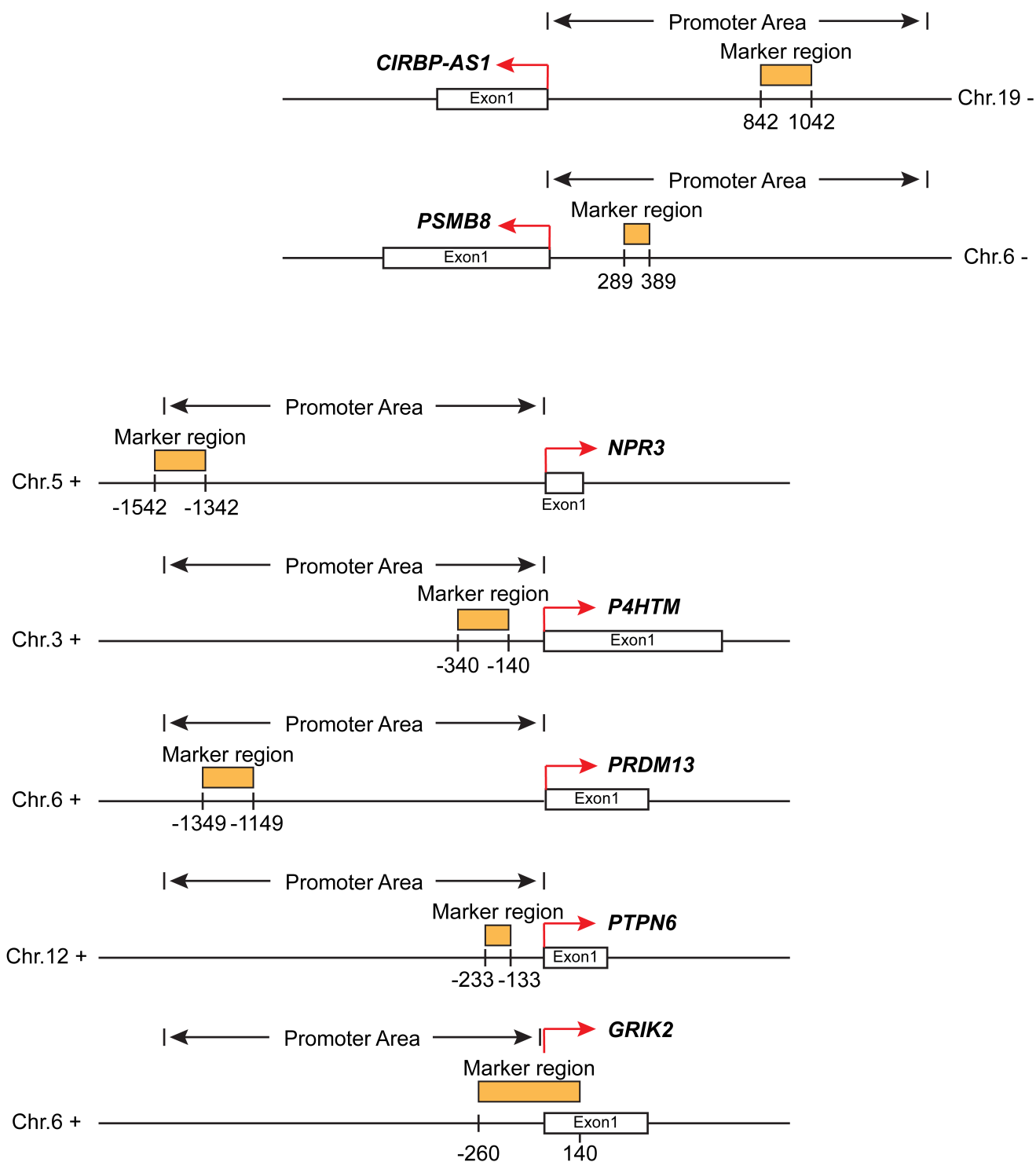

**Supplementary Figure S2.**

Diagram illustrating the genomic loci of the 7-DMR panel.

Supplement: Figure S2 — Supplementary Figure S2. Diagram illustrating the genomic loci of the 7-DMR panel. [file crc-23-0406-s05.pdf]
