# Supplementary material for: Comparative Epigenetic Profiling Reveals Distinct Features of Mucosal Melanomas Associated with Immune Cell Infiltration and Their Clinical Implications
Source: Cancer Res Commun. 2024 May 28;4(5):1351–62. doi: 10.1158/2767-9764.CRC-23-0406 (PMC11131765; doi:10.1158/2767-9764.CRC-23-0406)
Supplement: Figure S3 — Supplementary Figure S3. Heatmap depicting the association between methylation status of TERT and genes within 7-DMR panel and the immune cell infiltration in NEMM. [file crc-23-0406-s06.pdf]

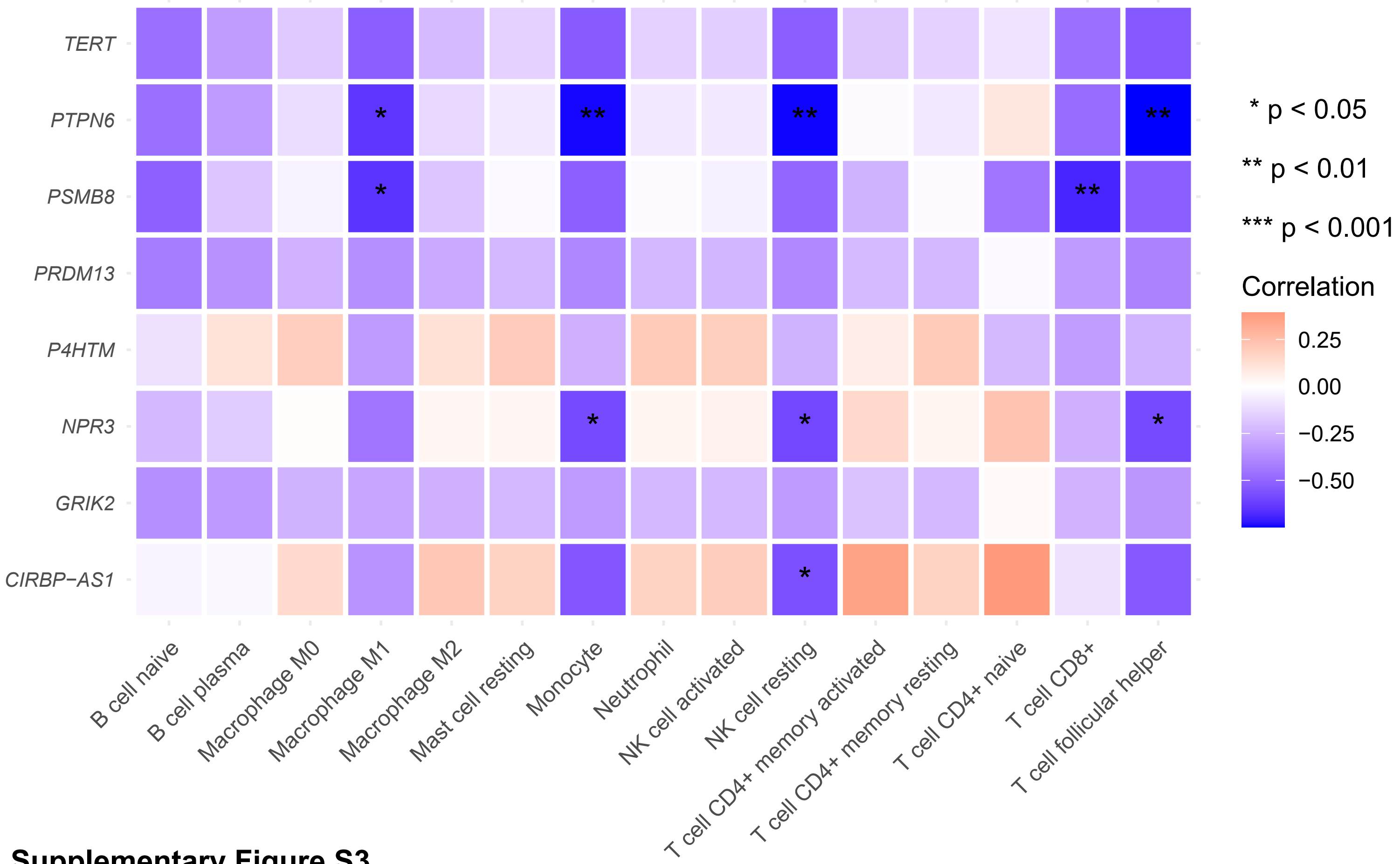

Supplementary Figure S3.

Heatmap depicting the association between methylation status of TERT and genes within 7-DMR panel and the immune cell infiltration in NEMM.
